# Patient experiences with general practice in Norway: a comparison of immigrant groups and the majority population following a national survey

**DOI:** 10.1186/s12913-020-05963-3

**Published:** 2020-12-01

**Authors:** Marte Kjøllesdal, Thor Indseth, Hilde Hestad Iversen, Oyvind Bjertnaes

**Affiliations:** Norwegian Institute of Public Health, Health Services Research, Postboks 222 Skøyen, 0213 Oslo, Norway

**Keywords:** Patient experiences, GP, Immigrants, Norway, National survey

## Abstract

**Background:**

Patient experience is an important indicator of quality of health care. In Norway, little is known about the quality of health care for immigrants. The aim of this study was to compare patient-reported experiences with general practice between the Norwegian-born population and immigrant groups.

**Methods:**

We performed secondary analyses of data from a national survey on patient experiences with general practice, including assessments of general practitioners (GPs) and their GP offices. The survey was carried out in Norway in 2018–19. The total number of respondents was 2029, with a response rate of 42.6%. Region of birth was available for 1981 participants, and these were included in the analyses (“Norway” (*N* = 1756), “Asia, Africa or South America” (*N* = 95), “Eastern Europe” (*N* = 70) and “Western Europe, North America or Oceania” (*N* = 60)). Five indicators of patient experiences were used as dependent variables in bivariate and multivariate analyses, with region of birth as the main exposure variable and other background variables about the patient as adjustment variables: “the GP” (measures related to communication and competency), “auxiliary staff” (politeness, competency, organization), “accessibility” (waiting times), “coordination” (with other services) and `enablement` (GP facilitates coping with/understanding illness).

**Results:**

Immigrants as a whole reported poorer experiences with general practice than the majority population, with significantly poorer scores on four of five patient experience indicators. Patients from Asia/Africa/South America reported poorer experiences than those from Norway on the indicators “GP”, “auxiliary staff”, “accessibility” and “coordination”: on a scale from 0 to 100 where 100 is the best, the difference ranged from 7.8 (GP) to 20.3 (accessibility). Patients from Eastern Europe reported lower scores on “GP” and patients from Western Europe/North America/Oceania reported lower scores on “auxiliary staff”. These associations were still significant after adjustment for sex, age, self-rated physical and mental health, number of contacts with the GP and education.

**Conclusions:**

For countries with a substantial proportion of foreign-born patients in the health system, immigrant background is an important parameter in quality improvement work. Immigrant background is also an important parameter in health service research.

**Supplementary Information:**

The online version contains supplementary material available at 10.1186/s12913-020-05963-3.

## Background

Patient experience is an important indicator of quality of health care, and may inform health personnel and policy makers about the strengths and weaknesses of health care delivery, and identify areas for improvement [1]. Improved patient experiences can translate into better communication, more informed and involved patients, more timely and correct diagnoses and stronger adherence to therapy [1]. Patient experiences have been related to clinical effectiveness and patient safety across a range of disease areas [1].

Patient experiences in general practice may vary over demographic characteristics of populations, including ethnicity and immigrant background. Several explanations for differences in patient-reported experiences could relate to both ethnicity and being an immigrant, such as differences in expectations of health care, and also in rating and reporting of experiences [2, 3]. Added to which, there may be differences in health care groups receive. Cultural barriers could make it harder to provide the same quality of care to all minority groups [4], and discrimination in services may occur [5]. Among relatively newly arrived immigrants, additional possible barriers to high quality care include language and lack of knowledge about the system [4].

Ethnic differences in patient-reported experiences in health care have been reported in the United Kingdom (UK), with Black, South Asian, and Chinese respondents assessing health care less favourably than White respondents [6]. Another study from the UK showed that Black and British White patients had the same satisfaction level [2]. Patients identifying as Vietnamese, however, reported higher satisfaction, but also lower expectations, in health care than Whites [2]. A limited number of European studies have looked into patient-reported experiences among immigrants. A study in 31 European countries reported consistently lower general satisfaction with general practitioners (GPs), among first generation immigrants than host populations [7]. In Germany, patients with a Turkish background (immigrants and their descendants) reported positive experiences of contact with their GP, with the exception of time spent in the waiting room, which was comparable to other patients in Germany [8]. In Sweden, the experience with primary health care was assessed among three groups of immigrants (from Chile, Turkey and Iran) [9]. Most patients felt that the GP understood their problem and respected their personality, culture and wishes, and they were satisfied with their consultation [9].

In Norway, studies suggest that immigrants use primary health care (PHC) services less often than native Norwegians, but that frequency of visits increases with length of stay [10]. It has also been found that some groups, especially from low- and middle-income countries use primary out-of-hours care more often than natives [11]. A study based on surveys conducted in 2000 and 2002 found that non-western immigrants were less satisfied with their primary care doctor than Norwegians [12]. However, the surveys were restricted to Oslo, only included a general satisfaction item, and did not distinguish between type of primary care doctor. Beyond this study, there is a lack of knowledge about patient experiences with general practice among immigrant patients in Norway.

From 2001, the GP scheme was introduced in Norway, with all citizens having the right to be monitored by one GP. The GP is the gatekeeper to most other health services in Norway. Equity in health services is a clear political goal in Norway. Immigrants are a group at risk of receiving poorer quality health care services than others, and there is a need to assess firstly whether immigrants actually do receive poorer quality services, and secondly in which areas and what could be done to improve services to this group. Recently, a report on patient experiences with general practice in Norway was published [13]. In the survey underlying this report, participants’ country of birth was assessed, and differences between immigrants and Norwegian-born were found in four out of five indicators of patient experiences. In this article, we aim to perform an in-depth assessment of differences in patient-reported experiences with general practice between Norwegian-born patients and immigrant patients. In the literature, differences in patient experiences are measured both according to various definitions of immigrant background and to ethnicity. We will study this among immigrants, defined as being born outside Norway. Patient satisfaction and patient experience are distinct concepts, the former representing a patient-reported outcome heavily influenced by both patients’ expectations and patients’ experiences with the structure and processes of health care [14]. In our study, the instrument primarily focus on patient experiences with the structure and processes of general practice, usually referred to as a patient-reported experience measure (PREM), but we also include an item about patient satisfaction.

## Methods

The national survey was commissioned by the Ministry of Health and Care Services, as part of an evaluation of the regular GP scheme in Norway, and conducted by the Norwegian Institute of Public Health (NIPH).

### Data sources

The national survey on patient experiences with GPs and GP practices was carried out in Norway in 2018–19 [13]. Eligible participants were contacted by mail, and could respond either on paper or electronically. Non-responders were sent two reminders. Patients were randomly selected from the lists of selected GPs. GP practices were stratified according to type of municipality and number of GPs in the practice, and randomly selected following the sampling plan. Participants were not offered any form of language assistance in relation to the survey. Hence, it is reasonable to assume that patients with inadequate language skills in Norwegian are underrepresented.

The questionnaire built on a previously used and validated instrument [15], with 17 additional questions related to the survey’s connection to the evaluation of the regular GP scheme (Supplementary material). The additional questions addressed issues around accessibility, coordination of services, user involvement and coping, and also background characteristics of patients, like immigrant background, education, health and contact with GP during the last year. A number of relevant user organizations were invited to contribute in the development of additional questions. Altogether 47 questions were included, and the updated questionnaire was tested with ten cognitive interviews. The 47 questions were grouped into the following thematic areas: “accessibility”, “review of the GP”, “auxiliary staff and organization”, “coordination”, “home visits”, “interpreters”, “payment”, “other concerns” and “background information”. There were also free-text fields for further comments from the participants, one related to experiences with the GP and the GP office, and another concerning their wishes for the future regular GP scheme.

### Variables

#### Dependent variables

Based on factor analyses of single questions, three indicators of patient experiences were established [13]: “the GP” (measures related to communication and competency), “auxiliary staff” (politeness, competency, organization), and “accessibility” (waiting times). Two indicators (“coordination” and “enablement”) were made based on theoretical considerations. “Coordination” included two questions on the coordination of health care services not included in the factor analyses because they were not relevant to many of the participants. “Enablement” was based on three questions (about being enabled to cope with/understand illness when in contact with the GP) not included in the factor analyses because they related to outcome, not experiences. All questions included in the indicators had the response categories “not at all”, “to a little extent”, “to some extent”, “to a large extent” and “to a very large extent”. These five response categories were scored 0, 25, 50, 75 and 100 respectively. Of the 47 questions, 19 are included in the indicators. The questions underlying each indicator are presented in Table 2. Six additional single questions were included: “How satisfied are you with your GP?”, “Is it difficult to reach the GP practice by phone?”, “Is it important for you to see your own GP when you have an appointment?”, “Do you experience having to wait in the waiting facilities beyond the scheduled time?”, “Does the communication between the GP and auxiliary staff protect your privacy?” and “Would you recommend your GP to friends or family?”

#### Independent variables

Self-reported characteristics of patients included region of origin (where were you born (four defined categories)?): “Norway”, “Asia, Africa or South America”, “Eastern Europe” and “Western Europe, North America or Oceania” (all except “Norway” categorized as immigrant background), sex, age, education, self-reported physical and mental health,, number of chronic diseases, and participant’s number of contacts with their GP/GP practice during the last 24 months.: “0”, “1”, “2–5”, “6–12”, “≥13”.

### Statistical analysis

Chi square was used to assess associations between region of origin and categorical variables, and ANOVA for differences in means between groups of region of origin. Multiple linear regression was carried out to assess associations between region of origin and patient experiences with GP (five indicators, overall satisfaction with GP and single questions not in indexes), adjusting for other background variables about the patients. Model 1 was adjusted for sex and age-group, model 2 was additionally adjusted for self-rated physical and mental health and having a chronic condition, and model 3 was additionally adjusted for number of contacts with the GP during the last 24 months and education. Correlation analysis (Pearson’s r) was used to assess which of the five aspects of patient experiences was most linked to general satisfaction with the GP and the practice for each region of origin.

We supplemented the linear regressions with multilevel regressions to check the potential effect of the GP practice level on regression coefficients and significance tests.

### Open-ended comments

The questionnaire included two free-text fields where participants were invited to say more about 1) their experiences with their GP and 2) what they wanted the GP scheme to be like in the future. The main objective of the qualitative analysis was to compare the comments from patients with immigrant background with the comments from the total sample published in the national report [13]. Accordingly, the same approach for qualitative analysis was applied for the data in this study as for the national reporting. Two researchers (MK and HHI) independently coded all responses from patients with immigrant background. Firstly, we aimed to determine whether the open-ended comments were of a positive, negative or neutral character. Neutral comments that did not address the GP services or give any specific evaluation of the health care quality were not included in the content analyses. Secondly, the most common themes and subgroups within the themes addressed by the patients were identified. Each open-ended comment was analysed systematically in an iterative manner by creating a thematic coding structure. When new themes emerged, the coding structure was revised and the previous comments re-read to determine congruence with new themes. Any coding ambiguities were resolved through discussion and joint agreement. Thirdly, sentiments and topics identified in the comments from patients with immigrant background were compared to the comments from the national sample.

## Results

A total of 4999 patients (aged > 16 years) were selected, of whom 201 could not be reached, 2 were dead and 36 actively declined to participate. Of the remaining 4760 patients, 2029 patients participated, which was a response rate of 42.6%. Of those, 48 had missing information on country of birth, and were thus excluded, leaving a sample of 1981 participants.

The proportion of female participants was slightly lower among immigrants from Western Europe/North America/Oceania than among participants from Norway, and all immigrant groups had a lower proportion in the highest age group than the Norwegian-born (Table 1). The proportion with good self-rated physical and mental health was lower among immigrants from Asia/Africa/South America than among participants from Norway (Table 1). The proportion of participants who had a proxy fill out the questionnaire was higher among immigrants from Asia/Africa/South America (8.4%, *N* = 8) and Eastern Europe (7.1%, *N* = 5) than among patients born in Norway (1.9%, *N* = 34) and Western Europe/North America/Oceania (1.7%, *N* = 1).

**Table 1:** Characteristics of participants according to participants’ region of birth

N (%)	Norway (*N* = 1756)	Asia/Africa/South America (*N* = 95)	Eastern Europe (*N* = 70)	Western Europe/North America/Oceania (N = 60)
Women	995 (56.7))	47 (49.5)	37 (52.9)	25 (41.7)*
Age (years)				
16–29	180 (10.3)	16 (16.8)	5 (7.1)	5 (8.3)
30–49	367 (20.9)	45 (47.4)	41 (58.6)	23 (38.3)
50–66	607 (34.6)	30 (31.3)	20 (28.6)	24 (40.0)
≥ 67	602 (34.3)	4 (4.2)	4 (5.7)	8 (13.3)
*Age, trend over groups*		***	***	**
Education				
Primary School	283 (16.2)	17 (18.1)	6 (8.6)	1 (1.7)
Secondary education	664 (38.1)	28 (29.8)	23 (32.9)	20 (33.3)
University or University College≤4 years	445 (25.5)	26 (27.7)	17 (24.3)	16 (26.7)
University or University College≥4 years	351 (20.1)	23 (24.5)	24 (34.3)	23 (38.3)
*Education, trend over groups*			*	**
Chronic disease	1131 (65.7)	52 (55.3)*	37 (53.6)*	32 (55.2)
Good self rated physical health	1233 (70.4)	22 (54.7)***	47 (67.1)	42 (70.0)
Good self rated mental health	1424 (81.3)	62 (66.0)***	54 (78.3)	49 (81.7)
Number of consultations last 24 months (N (min max))	11.0 (0–104)	8.3 (0–65)	7.4 (0–31)	8.9 (0–54)

Difference between group and Norwegian-born participants: *p < 0.05, **p < 0.01, ***p < 0.001

Mean scores for the five indicators, the questions underlying the indicators, and other single questions for the four groups are shown in Table 2. Patients from Asia/Africa/South America had lower scores than those born in Norway on the indicators “GP”, “auxiliary staff”, “accessibility” and “coordination.” They had lower scores on all questions included in these four indicators, except “GP provides sufficient information about use and side-effects of medication”. Patients from Eastern Europe had lower scores than those born in Norway on the indicator “GP”, with lower scores on six out of nine questions included. Patients from Eastern Europe also had lower scores than those born in Norway for the question on waiting times for acute consultations, but not for the indicator “accessibility”. Patients from Western Europe/North America/Oceania had lower scores on the indicator “auxiliary staff”. The lower scores on this indictor related to organization of the GP practice, and being met with respect at the reception, not to whether the staff were polite and competent. Immigrants from Asia/Africa/South America and Eastern Europe had lower scores than Norwegian-born patients for “conversation between GP and auxiliary staff protecting privacy”, and immigrants from Western countries had the highest scores on “time in waiting facilities beyond scheduled appointments”. There were no differences between non-immigrants and immigrants for general satisfaction with the GP. The indicators with the largest differences from Norwegian-born patients were accessibility (Asia/Africa/South America) and “conversation between GP and auxiliary staff protecting privacy” (Asia/Africa/South America and Eastern Europe), the latter with a score 28.9 points lower for Eastern Europe.

**Table 2:** Mean score (SD) on indicators and items according to region of birth

	Norway	Immigrants, all	Asia/Africa/South America	Eastern Europe	Western Europe, North America, Oceania
**GP**	79.0 (16.1)	73.3 (20.4)***	72.2 (19.6)**	72.9 (21.1)*	75.7 (21.0)
GP takes you seriously	84.0 (18.5)	75.5 (27.2)***	73.3 (28.2)***	75.0 (27.9)***	81.3 (24.2)
GP has enough time for you	74.4 (22.1)	70.1 (25.6)*	69.3 (26.3)*	69.0 (26.3)	73.1 (23.6)
GP talks so that you can understand her/him?	85.2 (16.8)	80.2 (22.9)***	79.2 (22.7)**	78.2 (25.8)**	84.1 (23.6)
GP is competent	82.7 (17.1)	77.8 (23.7)***	77.6 (22.8)*	77.8 (25.6)*	77.9 (23.3)
GP is interested in your situation	80.1 (19.8)	76.4 (24.5)*	75.6 (24.4)*	68.1 (26.1)	78.4 (24.8)
GP involves you as much as you want in decisions about you	79.8 (19.7)	72.5 (23.7)***	73.2 (22.8)**	68.1 (26.0)***	76.5 (21.9)
GP gives enough information about health problems and treatment	77.3 (20.4)	71.6 (26.0)***	72.1 (23.6)*	70.6 (28.5)*	71.9 (27.3)
GP provides sufficient information about use and side-effects of medication	65.0 (26.5)	63.8 (30.4)	60.9 (29.5)	70.2 (28.9)	60.5 (33.3)
GP refers you to a further examination or specialist when you think that is needed	82.2 (19.7)	75.0 (23.5)***	75.3 (19.7)**	72.9 (26.6)***	77.4 (25.2)
**Auxiliary staff**	79.0 (17.2)	72.0 (20.2) ***	68.2 (20.8)***	76.9 (18.5)	72.4 (20.3)*
Practice well organized	75.7 (19.9)	69.7 (22.3) ***	66.4 (23.1)***	74.2 (19.4)	69.5 (23.8)*
Auxiliary staff polite and competent	80.1 (18.7)	72.3 (21.9) ***	67.4 (23.1)***	76.6 (20.4)	75.0 (20.6)
Met with politeness and respect at reception	81.6 (19.3)	75.1 (22.0) ***	72.4 (22.2)***	79.5 (21.7)	74.5 (21.5)*
**Accessibility**	65.2 (27.1)	52.5 (30.0) ***	44.9 (30.1)***	57.3 (29.1)	58.8 (28.7)
Acceptability of waiting time for acute consultation	71.4 (29.6)	57.0 (33.8) ***	50.6 (33.8)***	59.9 (33.1)**	64.9 (33.2)
Acceptability of waiting time for normal consultation	59.7 (29.9)	48.4 (29.5) ***	40.2 (30.9)***	56.7 (27.2)	52.0 (26.6)
**Enablement**	65.4 (21.7)	64.7 (24.6)	66.5 (21.9)	64.0 (27.4)	62.6 (25.7)
Contact with GP helps you understand your illness	68.3 (22.8)	66.9 (24.4)	68.4 (21.1)	67.3 (26.3)	64.3 (27.5)
Contact with GP helps you cope with your illness	65.4 (23.5)	63.0 (26.6)	65.3 (25.0)	61.4 (28.4)	60.9 (27.2)
Contact with GP helps you keep yourself healthy	62.7 (24.6)	64.0 (27.5)	67.0 (25.3)	63.8 (30.1)	58.3 (28.0)
**Coordination**	75.0 (20.2)	68.7 (25.8) ***	63.8 (26.3)***	75.6 (20.9)	68.3 (29.5)
GP coordinates health services well	75.6 (20.5)	70.5 (25.7)**	67.5 (26.0)**	75.9 (21.0)	68.2 (30.4)
GP cooperates well with other services	75.0 (21.8)	68.7 (27.2)**	62.7(26.0)***	75.9 (23.1)	69.1 (30.4)
**Other questions**					
General satisfaction	82.7 (19.8)	77.0 (24.9) ***	77.0 (23.7)	76.6 (25.7)	77.3 (26.3)
GP practice difficult to reach by phone	72.1 (27.0)	70.5 (28.5)	67.1 (29.3)	75.0 (27.1)	71.4 (28.2)
Important to meet your own GP when you have an appointment	75.4 (24.9)	73.0 (30.5)	75.0 (29.9)	70.3 (31.6)	72.9 (30.2)
Have to wait in the waiting facilities after scheduled time	49.0 (25.9)	50.1 (27.2)	45.6 (29.0)	51.2 (27.9)	56.1 (21.9)*
Conversations with GP and staff protects your privacy	82.0 (21.1)	63.3 (34.5) ***	59.9 (32.8)***	53.1 (38.4)***	80.5 (25.9)
Recommend GP to friends/family	74.8 (26.1)	68.4 (31.5) **	66.5 (31.0)**	71.0 (32.2)	68.5 (33.0)

From analysis of variance (ANOVA). Difference between Norwegian-born participants and other groups: *p < 0.05, **p < 0.01, ***p < 0.001

The associations between indicators and region of origin found for unadjusted mean scores (in Table 2) were significant even when adjusted for sex, age, physical and mental health, number of contacts with the GP and education in regression models (except auxiliary staff among Western Europe) (Table 3), although adjustments slightly attenuated most associations. In addition to these, immigrants from Eastern Europe had lower scores than non-immigrants on accessibility in two of three regression models. Associations between region of origin and single questions not included in indexes also remained significant in fully adjusted models (Table 4). In addition, immigrants from Eastern Europe had lower scores for general satisfaction in the full model, and immigrants from Asia/Africa/South America when adjusted for sex and age only. Multilevel regressions with adjustments for the GP practice level gave the same overall results (results not shown here).

**Table 3:** Associations between region of birth and index for experiences with general practice. Reference category: Norway

	GP	Auxiliary staff	Accessibility	Enablement	Coordination
*B-coefficients and 95% confidence intervals*					
Immigrants, all					
Model 1	−5.79 (−8.24, −3.34)***	− 5.54 (− 8.10, − 2.98)***	−13.11 (− 17.16, − 9.05)***	− 0.47 (− 3.02, 2.89)	− 5.65 (− 8.99, 2.31)**
Model 2	− 5.00 (− 7.47, − 2.54)***	− 5.37 (− 7.99, − 2.76)***	−13.03 (− 17.17, 8.89)***	0.31 (−3.08, 3.69)	− 4.27 (− 7.27, − 0.91)**
Model 3	− 4.87 (− 7.33, − 2.42)***	−5.00 (− 7.61, − 2.40)***	− 12.80 (− 16.94, − 8.67)***	1.12 (− 2.43, 4.48)	−3.89 (− 7.24, 0.54)*
Asia/Africa/South America					
Model 1	−6.93 (− 10.41, − 3.45)***	− 9.09 (− 12.8, − 5.39)***	−20.95 (− 26.9, − 14.96)***	1.56 (− 3.27, 6.39)	− 10.44 (− 15.18, − 5.70)***
Model 2	−5.86 (− 9.44, − 2.34)**	−8.90 (− 12.6, − 5.11)***	−20.55 (− 26.67, − 14.46)***	2.64 (− 2.26, 7.53)	− 7.96 (− 12.75, − 3.17)**
Model 3	−5.51 (− 9.03, − 1.99)**	− 8.49 (− 12.26, − 4.72)***	−19.73 (− 25.84, − 13.61)***	3.58 (− 1.25, 8.41)	−7.36 (− 12.13, − 2.58)**
Eastern Europe					
Model 1	−6.30 (− 10.40, − 2.19)**	−0.64 (− 4.94, 3.67)	− 8.51 (− 15.29, − 1.73)*	−1.11 (− 6.81, 4.59)	1.12 (− 4.19, 6.44)
Model 2	−6.64 (− 10.77, − 2.52)**	− 0.86 (− 5.25, 3.52)	−9.03 (− 15.95, − 2.12)*	− 1.84 (− 7.54, 3.87)	0.88 (− 4.45, 6.21)
Model 3	−6.41 (− 10.51, − 2.32)**	−0.38 (− 4.73, 3.98)	−8.94 (− 15.82, − 2.05)*	−0.78 (− 6.41, 4.83)	1.40 (− 3.90, 6.70)
Western Europe, North America, Oceania					
Model 1	−3.11 (−7.62, 1.40)	− 5.43 (− 10.1, − 0.71)*	−6.48 (− 13.86, 0.90)	−2.70 (− 8.87, 3.47)	−6.33 (− 12.73, 0.08)
Model 2	−1.71 (− 6.21, 2.78)	−4.85 (− 9.62, − 0.07)*	−6.24 (− 13.80, 1.31)	− 0.85 (− 7.14, 5.38)	−4.93 (− 11.35, 1.49)
Model 3	−1.97 (− 6.45, 2.51)	−4.71 (− 9.47, 0.05)	−6.88 (− 14.40, 0.65)	−0.33 (− 6.47, 5.82)	−4.94 (− 11.34, 1.45)

From linear regressions. Model 1: adjusted for sex and age-group. Model 2: additionally adjusted for self-rated physical and mental health and having a chronic condition. Model 3: additionally adjusted for number of contacts during last 24 months and education

Difference between group and Norwegian-born participants: *p < 0.05, **p < 0.01, ***p < 0.001

**Table 4:** Associations between region of birth and single questions about experiences with general practice. Reference category: Norway

	General satisfaction	GP practice difficult to reach by phone	Important to meet your own GP when you have an appointment	Have to wait in the waiting facilities after scheduled time	Conversations with GP and staff protects your privacy	Recommend GP to friends/family
*B-coefficients and 95% confidence intervals*						
Immigrants, all						
Model 1	−5.58 (− 8.58, − 2.57)***	0.47 (− 3.58, 4.52)	−0.28 (− 3.87, 3.31)	4.09 (0.32, 7.85)*	− 19.57 (− 23.10, − 16.03)***	− 6.34 (− 10.46, − 2.23)**
Model 2	−4.58 (− 7.62, − 1.53)***	1.10 (− 3.01, 5.22)	− 0.49 (− 4.11, 3.13)	4.73 (0.88, 8.58)*	− 19.28 (− 22.83, − 15.74)***	− 5.49 (− 9.68, − 1.30)*
Model 3	−4.11 (− 7.13, − 1.09)**	1.36 (− 2.77, 5.48)	0.28 (− 3.30, 3.86)	5.14 (1.28, 9.00)**	− 19.67 (− 23.21, − 16.13)***	−4.86 (− 9.03, − 0.70)*
Asia/Africa/South America						
Model 1	−5.46 (− 9.80, − 1.12)*	−2.50 (− 8.30, 3.30)	2.40 (− 3.18, 7.78)	0.49 (− 5.06, 6.03)	−23.29 (− 28.22, − 18.37)***	− 8.20 (− 14.12, − 2.28)**
Model 2	−4.30 (− 8.70, 0.11)	− 1.48 (− 7.38, 4.42)	1.42 (− 3.84, 6.69)	1.49 (− 4.18, 7.17)	−22.66 (− 27.60, − 17.72)***	−7.47 (− 13.52, − 1.42)*
Model 3	− 3.73 (− 8.09, 0.63)	−1.56 (− 7.48, 4.36)	2.08 (− 3.12, 7.29)	1.74 (− 3.93, 7.40)	− 22.46 (− 23.21, − 16.13)***	−6.61 (− 12.61, − 0.61)*
Eastern Europe						
Model 1	−5.92 (− 11.00, − 0.84)*	5.09 (−1.91, 12.08)	3.20 (− 9.21, 2.82)	5.39 (− 1.08, 11.86)	−30.07 (− 35.90, − 24.25)***	−3.79 (− 10.84, 3.25)
Model 2	−6.08 (− 11.29, − 0.95)*	5.40 (−1.71, 12.50)	−3.26 (− 9.28, 2.77)	5.78 (− 0.82, 12.38)	−30.53 (− 36.34, − 24.72)***	−4.01 (− 11.11, 3.10)
Model 3	−5.50 (− 10.57, − 0.42)*	6.02 (−1.08, 13.12)	− 2.13 (− 8.03, 3.78)	6.34 (− 0.26, 12.94)	−31.18 (− 36.98, − 25.37)***	−3.05 (− 10.10, 4.01)
Western Europe, North America, Oceania						
Model 1	−5.09 (− 10.54, 0.37)	0.43 (− 7.27, 8.13)	− 0.59 (− 7.03, 5.85)	8.58 (1.72, 15.45)*	− 2.17 (− 8.17, 3.84)	−5.79 (− 13.24, 1.66)
Model 2	−3.32 (− 8.83, 2.18)	0.64 (− 7.13, 8.41)	−0.05 (− 6.49, 6.39)	8.95 (2.00, 15.90)*	−1.93 (− 7.87, 4.01)	−3.82 (− 11.41, 3.77)
Model 3	− 3.16 (− 8.62, 2.30)	0.88 (− 6.90, 8.66)	−0.66 (− 5.69, 7.01)	9.48 (2.51, 16.44)**	− 2.87 (− 8.79, 3.05)	−3.57 (− 11.11, 3.98)

From linear regressions. Model 1: adjusted for sex and age-group. Model 2: additionally adjusted for self-rated physical and mental health and having a chronic condition. Model 3: additionally adjusted for number of contacts during last 24 months and education. Difference between group and Norwegian-born participants: *p < 0.05, **p < 0.01, ***p < 0.001

All five indicators of patient experiences were significantly correlated with general satisfaction with the GP in all groups of country of origin (Table 5), with strongest correlations for the indicator “GP” and weakest correlations for “accessibility”. The importance of each indicator varied somewhat between groups. “Auxiliary staff” had a weaker correlation with general satisfaction among patients from Eastern Europe than those born in Norway. “Enablement” was more important for general satisfaction among all groups of immigrants than among those born in Norway. “Coordination” had a weaker correlation with general satisfaction among immigrants from Asia/Africa/South America and a stronger correlation among immigrants from Eastern Europe.

**Table 5:** Correlation between five indicators of patient satisfaction and general satisfaction with GP

	GP	Auxiliary staff	Accessibility	Enablement	Coordination
Norway	0.81***	0.48 ***	0,40 ***	0,66 ***	0,66***
Asia/Africa/South America	0.78 ***	0.36***	0,36**	0,74***	0,54***
Eastern Europe	0.86***	0.37***	0,37**	0,79***	0,69***
Western Europe, North America, Oceania	0.85***	0,30***	0,30*	0,78***	0,79***

Pearson’s correlation coefficient

*p < 0.05, **p < 0.01, ***p < 0.001

Bold numbers: correlations significantly different from Norway, tested by interaction in linear regressions

The free-text fields for further comments were used by 54 (24%) patients with immigrant background. Regarding experiences with the GP/GP practice, 40% were positive, 20% were negative, 22% were neutral and 18% both positive and negative. The content analysis identified the following themes or domains most commonly addressed: accessibility, communication and relational aspects and professionalism. The positive comments most commonly expressed general satisfaction with the GP. The negative comments mostly regarded long waiting times and also poor communication. One participant commented “My doctor is always 45 minutes late, and is always in a hurry.” Another wrote “My doctor never gives me any explanation what to do. I feel visiting the doctor gives me very little every time I am there.” When asked about wishes for the GP scheme in the future, some wanted it to continue as it is, and a few mentioned better communication with patients and improvement in waiting times. In comparison, 34% (*N* = 693) of the total sample provided an open-ended comment, of which 32% were positive, 25% were negative, 30% were neutral and 13% were both positive and negative [13]. The themes most commonly addressed were the same as among immigrant patients.

## Discussion

Immigrants from Asia/Africa/South America scored the GP and GP practice significantly poorer than non-immigrants on most patient experience indicators. Immigrants from Eastern Europe were more similar to non-immigrants, but scored the GPs lower on the GP indicator and the single items on accessibility for acute consultations, protection of privacy and overall satisfaction with the GP (adjusted model). Immigrants from other parts of the world had experiences comparable to those born in Norway in most areas, except lower score on organization of GP practice and being met with respect at the reception.

Previous studies have reported differences according to immigrant background, and also ethnicity, in patient experiences and general satisfaction with the GP [7–9], in line with our results. Low scores among immigrants from Asia/Africa/South America on items related to communication (such as GP talking so that you can understand him/her) correspond to difficulties in communication with the GP reported by Iranian, Turkish and Chilean immigrants in Sweden [9], and among South Asian and Chinese respondents in the UK [6]. Poor communication was also a main domain raised by patients in free-text fields in the current study. Barriers related to language and communication in primary care are expressed in previous qualitative studies, e.g. among asylum seekers and refugees in the UK [5].

Accessibility was another main issue raised by participants filling out the free-text fields, and patients from Asia/Africa/South America reported low scores on accessibility and waiting time. This corresponds to poor experiences with accessibility and waiting time reported among Turkish patients in Germany [8]. Immigrants from Eastern Europe had lower scores than patients born in Norway for the GP and for accessibility of acute consultations. Previous studies among Polish immigrants in Norway have reported challenges related to communication and language, and poor experiences with examinations and treatment [16].

Immigrants’ poor experiences in the health care system may be explained both by factors related to the immigrants and by factors in the health care system. Potential barriers within the health care system to high quality care to immigrants include discrimination [5], insufficient use of interpreters and limited knowledge among health workers about challenges that immigrants face. In quality improvement work, both factors related to immigrants and factors in the health care system are possible entry points for intervention. The results of the current study point towards the importance of language and communication. Interventions targeting the immigrant community could include implementing and testing incentives or programmes to improve host language skills and health literacy. Within the health services, one could implement and test programmes and incentives aimed at improving service quality for immigrants, e.g. by abating negative effects of poor language skills and poor health literacy. Raised awareness about immigrants’ poorer experiences in the health care system, the role of health workers and potential discrimination in the health care system, is an important part of improving experiences among immigrants. A systematic review in the US showed an association between cultural competence training of health personnel and patient satisfaction among minority groups [17]. Measuring outcomes and experiences among immigrants might have an effect on awareness in itself. Norway has a clear political goal regarding equity in health services, and there are no substantial legal or economic barriers to access to health care. This indicates a need to work with quality improvement work from within the health care system.

Challenges especially important to some immigrants groups may include poor skills in the host language, low health literacy, lack of knowledge about the health system in the host country and cultural differences. Further, differences in expectations have been suggested as one reason for differences in patient-reported experiences [2, 18]. For example, patients not knowing the guidelines on limiting the use of antibiotics may interpret a GP’s refusal to prescribe antibiotics differently from patients who are familiar with this goal. In a qualitative study among Pakistani women in Norway, participants opined that Norwegian doctors do not prescribe medicines as often as they would like [19]. Poor experiences with the health care system among Polish immigrants in Norway have been related to a more paternalistic GP role in Poland than in Norway [16]. It has been suggested that unfamiliarity with the Norwegian system, including rationales behind the treatment provided, correlates with low confidence in the system in this group. Previous studies have suggested lower expectations to the health system to explain higher satisfaction in some minority groups [2]. Immigrants in our sample were more highly educated than the general immigrant population in Norway, and we speculate that this relate to higher expectations, and thus lower satisfaction. The Norwegian-born in our study were also more highly educated than the general Norwegian population, thus should apply also to this group, and not so much to the differences between immigrants and Norwegian-born.

### Strengths and limitations

This is the first study to explore patient experiences with the GP and GP practice in a national survey in Norway, in which experiences can be assessed according to region of origin. The questionnaire has been developed according to established procedures, as part of national measurements of health care quality [13]. It was not however developed to fit, or tested among, immigrant populations, and we therefore do not know how the questions are interpreted by participants of various cultural and lingual backgrounds. Previous research have suggested that Asians’ low extreme response tendency may explain ethnic differences seen in patient experiences [3]. Other have demonstrated no differential item functioning and measurement equivalence between Whites and Asians in responses to experience scales [20, 21], suggesting that lower scores among Asians reflect differences in care [22]. No special measures were taken to include immigrants in the survey, as this was not one of its original aims.

There is a general tendency that persons with disadvantaged socioeconomic position, including low educational levels and low income, as well as immigrants, are underrepresented in Norwegian health surveys, and we expect this to occur in the present study as well. Moreover, we expect immigrants who are most distant from the health care system (i.e. low users) is particularly underrepresented. Compared to national numbers, the proportion of participants with primary education was low, and the proportion with higher university degrees was high in this study. The proportion of immigrants in the survey was 11%, compared to 14% in the population.

A larger proportion of participants from Asia/Africa/South America and Eastern Europe than others reported having had their questionnaire filled out by a proxy, but well above 90% responded themselves. We do not have information about who these proxies were, but previous research suggests that they are most often close family members [23]. Research has also shown that answers from proxies differ only slightly from patients’ own answers [24]. As the questionnaire was provided in the Norwegian language only, we have to assume that there is a selection of participants with reasonably fair Norwegian skills. We do not know whether language skills are associated with patient experiences with the GP in this study. One study among Turkish patients in Germany showed poorer experiences among patients with good or excellent German skills than among others [8], but this is solely one study and the generalizability to our context is uncertain. However, a link between language skills and the probability of non-response seems plausible; thus we could base our expectation on experiences with non-response modelling in the national patient experience program in Norway. This knowledge base shows slightly poorer scores when accounting for non-response.

The categories of region of origin were very broad, and include a large number of countries which probably vary in a range of measures, such as expectations of the Norwegian health system, health, cultural and linguistic backgrounds, reason for migration, socio-demographic factors and length of stay in Norway. Adjustments for age, sex, self-reported health and number of contacts with the GP slightly attenuated differences between groups, but made no notable changes to conclusions. In addition to more homogenous categories, we stress the importance of larger samples in future studies to improve precision and to achieve more robust sub-group analysis (for instance, immigrant group by gender, country of birth and age).

Open-ended comments were provided by a smaller proportion of respondents with immigrant background than other respondents. Content analysis identified common themes, but a higher proportion of the respondents with immigrant background gave comments classified as positive.

## Conclusions

Immigrants, and especially immigrants from Asia, Africa and South-America, reported poorer patient-reported experiences than the majority population. For countries with a substantial proportion of foreign-born patients in the health system, and with an aim to achieving equity in health services, immigrant background is an important parameter, both in research and in quality improvement work and monitoring. The results suggested that countries with large economic and cultural distances to the host country experienced the largest gap in patient-reported experiences compared to the host population.

## Supplementary Information

**Additional file 1.**

## Data Availability

The datasets generated during and/or analysed during the current study are available from the corresponding author on reasonable request.

## Abbreviations

GP: General Practitioner
PHC: Primary Health Care
