# Supplementary material for: Patient experiences with general practice in Norway: a comparison of immigrant groups and the majority population following a national survey
Source: BMC Health Serv Res. 2020 Dec 1;20:1106. doi: 10.1186/s12913-020-05963-3 (PMC7708102; doi:10.1186/s12913-020-05963-3)
Supplement: Supplementary file 1 — Additional file 1. [file 12913_2020_5963_MOESM1_ESM.docx]

Additions to questionnaire in:

Holmboe O, Iversen HH, Danielsen K, Bjertnaes O. The Norwegian patient experiences with GP questionnaire (PEQ-GP): reliability and construct validity following a national survey. 2017;7(9):e016644.

7. Do you usually see your own GP when you have an appointment?

8. Is it important for you to see your own GP when you have an appointment?

17. Does your GP print out a list of your medicines when you change medication?

23. Note minor changes in the wording. (2014: Do you feel that your GP is good at coordinating the range of health services available to you? 2018: Do you feel that your GP is good at coordinating

the health services available to you?)

25. Does your GP refer you for further investigation or to a specialist when you think you need that?

26. Have you ever needed a home visit from your GP?

27. Think of the last time you needed a home visit. Did you receive one?

28. Have you ever needed an interpreter at the GP’s?

29. Think of the last time you needed an interpreter. Were you offered one?

30. How do you normally pay for the consultation with the GP?

By card at reception

Using a self-service payment terminal

By invoice

By cash

Other

Not applicable

31. How would you rather pay for the consultation with the GP?

33. Overall, how would you assess your GP?

Much worse than expected

Somewhat worse than expected

As expected

Somewhat better than expected

Much better than expected

*Not applicable*

38 When were you last in contact with your GP/GP surgery?

Less than 1 month ago

1-3 months ago

4-6 months ago

7-12 months ago

More than 12 months ago

*Not applicable/Don’t know*

39. How many times have you been in contact with your GP or GP surgery in the last 12 months?

0 times

Once

2-5 times

6-12 times

13 or more times

40. What are the reason(s) why you have not had contact with your GP or GP surgery in the last 12 months?

No need

Bad experiences with the GP/GP surgery

Not available when it suits me

Use company health service

Use private health service (e.g. Volvat,

Aleris)

Use other public health services (e.g.

emergency medical centre, district psychiatric centre)

Too expensive

Long journey

Not had time

Reluctant to make contact for other

reasons than mentioned above

Other

If you wish, you can write more about why you have not had contact with your GP or GP surgery the last 12 months:

41. How would you assess your physical health?

Very poor

Rather poor

Both-and

Rather good

Very good

42. How would you assess your mental health?

Very poor

Rather poor

Both-and

Rather good

Very good

44. Do you have any of the following long-term health problems/conditions?

1. High blood pressure (hypertension)
2. Heart disease, including myocardial infarction
3. Diabetes
4. Asthma, or chronic lung diseases such as chronic bronchitis, emphysema or COPD
5. Depression, anxiety or other mental health problems
6. Substance abuse problems
7. Cancer
8. Musculoskeletal ailments, including joint pain or arthritis
9. Have suffered a stroke
10. Have other long-term health problems or conditions
11. Have no long-term health problems/conditions

45. What is your highest level of educational attainment?

Primary school

Upper secondary school

College/university (0-4 years)

College/university (4 years or more)

46. Where are you born?

1. Norway
2. Asia (incl. Turkey), Africa or South America
3. Eastern Europe (all countries regardless of EU membership)
4. Western Europe, North America or Oceania
